# Pharmainnovationen: überragende Position der USA und Schwächen der deutschen Forschung

**DOI:** 10.1007/s10273-021-2985-3

**Published:** 2021-08-24

**Authors:** Andreas Eckert, Wolfgang Maennig

**Affiliations:** 1 Frühphasenfinanzierung GmbH, Eckert Wagniskapital und, Friedenstraße 2, 16341 Panketal OT Zepernick, Deutschland; 2 Fakultät für Wirtschafts- und Sozialwissenschaften, Universität Hamburg, Von-Melle-Park 5, 20146 Hamburg, Deutschland

**Keywords:** H51, I23, O32

## Abstract

Die Innovationskraft von Forschungsleistungen wird oft an Inputs wie Forschungsmitteln oder Outputs wie Patentanmeldungen gemessen. Hier wird ein neuartiger Indikator für pharmazeutische Innovationskraft vorgestellt, der sich auf die weltweiten medizinischen Durchbrüche und die assoziierten Patente konzentriert. Demnach sind US-Unternehmen von 2010 bis 2019 für 55 % der weltweiten medizinischen Durchbrüche verantwortlich, ihre deutschen Konkurrenten für rund 9 %. Bei den zugrundeliegenden Ankerpatenten ist die Dominanz der USA mit 62 % noch größer — aus Deutschland kommen nur 7 % der Ankerpatente. US-Universitäten halten 3,8 % aller Ankerpatente — deutsche Universitäten keine. Die Schwäche der deutschen Universitäten kann nicht durch die deutschen außeruniversitären Forschungsinstitute ausgeglichen werden.

## Abstract (en)

The innovative strength of research performance is often measured in terms of inputs such as research funds or outputs such as patent applications. We present a novel indicator of pharmaceutical innovativeness that focuses on global medical breakthroughs and associated patents. According to this indicator, US companies account for 55 % of global medical breakthroughs from 2010 to 2019, and their German competitors account for about 9 %. In terms of underlying anchor patents, the dominance of the US is even larger, at 62 %, while only 7 % of anchor patents come from Germany. US universities hold 3.8 % of all anchor patents; German universities hold none. The weakness of German universities cannot be compensated by German non-university research institutes.

Bei der Entwicklung von Impfstoffen gegen COVID-19 spielen in Deutschland beheimatete Unternehmen (Biontech und eventuell Curevac) eine führende Rolle. Bei aller Freude über ihre Beiträge dürfen sie nicht den nüchternen Blick auf die relative internationale Leistungsfähigkeit der deutschen Pharmaforschung verstellen. Wir berechnen die Innovationskraft im Pharmabereich, indem wir – unseres Erachtens erstmalig – die Zulassungen von Neuen Molekularen Einheiten (NME) bei der US-amerikanischen Food and Drug Administration (FDA) und die damit assoziierten Patente untersuchen.

Die assoziierten Patente sind als Gradmesser für die Qualität von Forschung und Entwicklung (FuE) besonders geeignet, besser als Inputfaktoren wie Forschungs- und Entwicklungsausgaben, besser auch als die Zählung von Patentanmeldungen und selbst besser als Patentregistrierungen. Die meisten registrierten Patente bleiben ungenutzt; 10 % der wertvollsten deutschen Patente machen mehr als 80 % des Wertes aller Patente aus (Scherer und Harhoff, 2000). Es ist deshalb nur bedingt zielführend, die Patente einer Firma oder eines Landes zu zählen, ohne auf ihren Wert zu achten (Nagaoka et al., 2010).

Wir wählen die pharmazeutische Forschung, da sie derzeit im Fokus der öffentlichen Aufmerksamkeit und der Forschungsförderung steht. Schon in „normalen“ Zeiten sind die patentfähigen Innovationen dieser Industrie von hoher Relevanz für das menschliche Wohlbefinden, weil sie ex definitione durch Krankheitsheilungen und/oder Lebensverlängerungen unmittelbaren Nutzen stiften (Grabowski, 2002). Zudem hat die Pharmaforschung in Deutschland eine historische Bedeutung; lange galt Deutschland als „Apotheke der Welt“. Diese Position ist verloren gegangen, wie unsere Berechnungen zeigen. Deutschland und seine Entscheidungsträger:innen sollten sich Gedanken machen, wie die alte Stärke zumindest ansatzweise wieder gewonnen werden kann.

## NME und verknüpfte Patente als Innovationsindikatoren in der Pharmazie

Für die Analyse ziehen wir die NME aus den Statistiken der FDA heran. Dort wird zwischen signifikant innovativen Medikamenten (NME) und lediglich inkrementellen Verbesserungen unterschieden. Als NME gelten ausschließlich neuartige chemische Verbindungen oder auch Einnahmefor-

* Für kritische Hinweise danken wir Leander Fortmann, Detlev Ganten, Martin Stratmann und Albert Rupprecht, die nicht alle Positionen teilen. Wir danken Nadine Schaarschmidt für exzellente Forschungsassistenz. Verbleibende Fehler gehen zulasten der Autoren.

**Dr. Andreas Eckert** ist Vorsitzender des Förderfonds Wissenschaft in Berlin.

**Prof. Dr. Wolfgang Maennig** ist Professor für Wirtschaftspolitik an der Universität Hamburg.

men, die Patient:innen neue Behandlungsmöglichkeiten eröffnen (FDA, 2020). Z. B. gehören Antikörper, die an bislang unbekannte Epitope binden, zur Kategorie der NME – Variationen bekannter Medikamente hingegen nicht.

Im Untersuchungszeitraum von 2009 bis 2019 wurden – trotz der weltweit hohen Forschungsleistungen – lediglich 378 NME registriert, weniger als 40 pro Jahr. Da heute fast ausschließlich zugelassene Medikamente die Patient:innen erreichen, repräsentieren die NME den harten Kern des weltweiten medizinischen Fortschritts. Sämtliche NME im untersuchten Zeitraum sind durch Patente geschützt – im FDA-Zulassungsbescheid wird detailliert auf die Schutzrechte Bezug genommen. Der Sachverhalt ermöglicht es, diejenigen Forschenden und Erfinder:innen zu identifizieren, die direkt zur Entwicklung von NME beigetragen haben. Mögen andere auch Hilfe geleistet haben, so waren diese für die Zulassung nicht wesentlich. Im Gegenteil: Aufgrund ihrer Nicht-Patentierungen verbauten sie sich die Chance, ihre Innovation zur Zulassung zu bringen. Ohne Patent keine Investierenden, ohne Investierende keine Medikamente.

Wirtschaftlich sind NME attraktiv, da sie in der Regel den Weltmarkt adressieren. Es gibt kaum Zulassungen für Medikamente, die nicht bei der FDA beantragt werden, da die USA als lukrativster Markt und die FDA als weltweit wichtigste Arzneimittel-Zulassungsbehörde angesehen werden. Es finden sich aber auch kaum Medikamente, die ausschließlich bei der FDA eingereicht werden. Vielmehr reichen die Pharmakonzerne ihre Dossiers weltweit ein. Was in der Statistik der FDA auftaucht, findet sich mit kurzer Verzögerung bei der Europäischen Arzneimittelbehörde (European Medicines Agency, EMA) oder umgekehrt. Der Grund hierfür liegt einerseits in den hohen Kosten für klinische Prüfungen, die Konzerne dazu zwingen, alle Vermarktungsmöglichkeiten zu nutzen, andererseits in den relativ geringen (Grenz-)Kosten einer Registrierung bei den zuständigen Behörden. Zwischen den Anmeldungen bei FDA und EMA besteht in 90 % der Fälle Deckungsgleichheit.

Die FDA-Statistiken zu den NME haben für die Ermittlung der relativen Leistungsfähigkeit einer Volkswirtschaft eine Reihe von herausragenden Eigenschaften. Sie kategorisieren die Zulassungen nicht nur hinsichtlich ihrer Erfindungshöhe, sondern benennen im „Orange Book“ zugleich alle wesentlichen Details, die in den Zulassungsantrag eingeflossen sind, inklusive des Hauptsitzes des Zulassungsinhabers (in der Regel ein Pharmaunternehmen). Firmen sind gesetzlich gehalten, innerhalb von 30 Tagen nach Erhalt einer Zulassung detaillierte Patentinformationen zur Verfügung zu stellen. Über die Patentansprüche werden verlängerte Exklusivitätsperioden festgelegt, die etwa Medikamente für seltene Krankheiten (*orphan drugs*) oder pädiatrische Arzneimittel erhalten. Da es um große Finanzvolumen geht, werden die Angaben sorgfältig zusammengestellt und erlauben dadurch einen hochwertigen Vergleich der verschiedenen nationalen Wissenschaftssysteme im Bereich der Medizin- bzw. Pharmaforschung.

Bei der Erstellung des Datensatzes zu NME-Anmeldungen und den verknüpften Patenten folgen wir Sampat und Lichtenberg (2011). Aus dem Orange Book entnehmen wir die Verknüpfung der NME-Anmeldungen mit den Nummern der Patente, auf die sich die Zulassungen stützen. Diese bezeichnen wir im Einklang mit der Literatur als Ankerpatente (*anchor patents*). Ferner berücksichtigen wir Trajtenberg et al. (1997) folgend die Elternpatente, d. h. die Zitationen in den Ankerpatenten. Abbildung 1 zeigt den Zusammenhang von NME, Anker- und Elternpatenten.

Die verwendeten Vorwärtszitate von Patenten sind – wie jeder Qualitätsmaßstab – mit Problemen behaftet, die jedoch beim Vergleich mit alternativen Qualitätsmaßstäben hinnehmbar sind (Grimaldi et al., 2015; de Saint-Georgesa und van Pottelsberghe de la Potteriea, 2014; Nagaoka et al., 2010; OECD, 2008). So benötigen Patentnutzungen und -zitierungen Zeit, sodass der von uns verwendete Indikator unter einem Trunkierungsproblem leiden könnte: Jüngere Patente realisieren eine kleinere Zahl von Nutzungen und Zitierungen. Allerdings erfolgen mehr als 50 % der Zitierungen eines Patents innerhalb der ersten fünf Jahre (OECD, 2008). Und in unserem Fall erfolgt insofern eine Ex-post-Evaluierung, als dass wir die Nutzung durch eine NME voraussetzen und sämtliche dort genutzten Patente berücksichtigen, ungeachtet ihres Alters.

## Daten, Methodik und Ergebnisse

Zwischen 2009 und 2019 wurden von der FDA 378 NME zugelassen, denen 1.392 Ankerpatente und 30.317 Elternpatente zugrunde liegen. Abbildung 2 zeigt eine tendenziell steigende Zahl von NME-Anmeldungen in den letzten Jahren. Die NME von 2019 sind nicht in der Abbildung enthalten, da Patentmeldungen erst in die Statistik eingehen, wenn die Patentdokumente vollständig veröffentlicht werden – was aus Schutzgründen meist erst 1,5 Jahre später erfolgt. Ausgehend vom Sitz der Anmeldenden kommen von den 378 NME 207 Anmeldungen aus den USA, 34 aus Deutschland, 26 aus der Schweiz, 18 aus Japan, 4 aus Spanien, 16 aus den Niederlanden, 18 aus Irland, 11 aus dem UK und 9 aus Kanada. Zu den stärksten NME-Unternehmen gehören die Novartis AG (CH, 14), Portola Pharmaceuticals, Inc. (USA, 13), Janssen Oncology, Inc. (USA, 12) Genentech, Inc. (USA, 11) und Allergan Holdings Unlimited Company (IE, 9) sowie von deutscher Seite die Bayer AG (8) und Boehringer Ingelheim Pharma KG (7).

Abbildung 1

**Bezug zwischen innovativen Medikamenten (NME) und den Patentgenerationen**

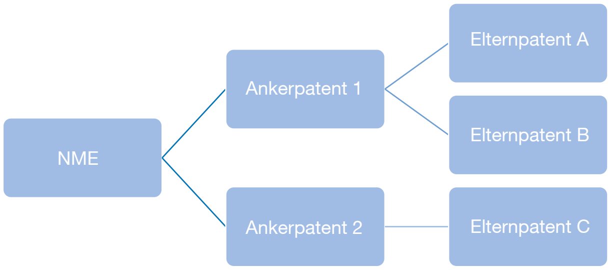

Quelle: eigene Darstellung.

Abbildung 2

**Zahl der Anmeldung von NME bei der Food and Drug Administration (FDA)**

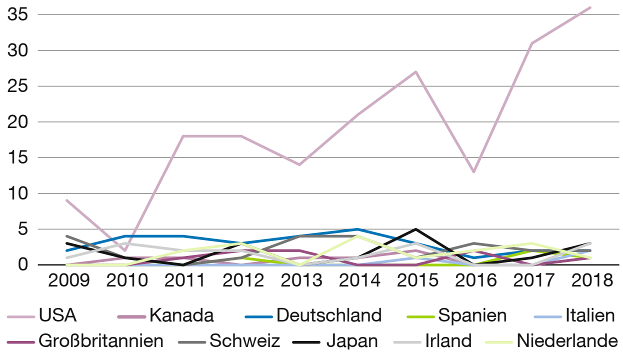

Quelle: eigene Darstellung auf Basis von FDA.

Um über die Nummer der Ankerpatente aus dem Orange Book weitere Informationen zu den Anmeldern und deren Herkunft zu erlangen, wurde mit R ein Webscraping programmiert, das diese Informationen aus der Patentdatenbank DEPATISnet ausliest. Auch bei den Ankerpatenten zeigt sich eine steigende Tendenz (vgl. Abbildung 3); die Einschränkung der Aussagekraft der Anmeldungen am Ende des Betrachtungszeitraums gilt auch hier.[1] Der Großteil der 1.392 Ankerpatente stammt aus den USA (860). Zweithäufigstes Anmeldeland ist Japan (117), Deutschland nimmt mit 97 Innovationen Rang drei ein. Allein 49 Ankerpatente sind der Firma Boehringer Ingelheim zuzurechnen, die damit weltweit Rang 1 in der Liste der ankerpatentanmeldenden Institutionen einnimmt. Unter den Institutionen befindet sich mit Bayer eine weitere deutsche Institution unter den Top 10. 53 Ankerpatente wurden von US-Universitäten angemeldet. Von deutschen Universitäten wurden keine Ankerpatente angemeldet; acht Patente wurden von deutschen außeruniversitären Forschungsinstituten hervorgebracht. In Japan kamen ausschließlich Patentanmeldungen von Pharmafirmen. Dieses Ergebnis mag daran liegen, dass 43 Ankerpatente nicht in lateinischer Schrift eingetragen waren und durch unser Webscraping-Programm nicht ausgelesen werden konnten. Pro Kopf der Bevölkerung sind von den USA 2,62 Patente pro Mio. US-Einwohner:innen angemeldet worden, in Deutschland 1,17 und in Japan 0,93 Patente.

Um die Ankerpatente mit den zugehörigen Elternpatenten zu verknüpfen, nutzten wir zunächst Google Patents. Sodann ermittelten wir mit unserem Webscraping-Programm die Anmelder und deren Herkunft aus der Patentdatenbank DEPATISnet. Die ausgelesenen Daten wurden einem Screening unterworfen. Insgesamt werden 30.317 Elternpatente von den Ankerpatenten zitiert. Aus den USA stammen 13.731 der Elternpatente (45 %). Hiervon entfallen 1.018 auf US-Universitäten.[2] Deutschland liegt mit 2.573 Elternpatenten hinter Japan mit 2.845. Bei 21 Elternpatenten ist eine deutsche Universität beteiligt; bei 49 sind außeruniversitäre Forschungsinstitute involviert. Japanische Universitäten sind bei sieben Elternpatenten (mit-)verantwortlich.

Es kann festgehalten werden, dass die USA sowohl bei den NME, als auch bei den Anker- und Elternpatenten dominieren. Deutschland fällt bei den NME und bei den Patenten deutlich zurück. Dies gilt bei Betrachtung der absoluten Zahlen sowie bei Relativierung anhand der Bevölkerungsgrößen – nicht hingegen anhand des Bruttoinlandsprodukts (BIP) (vgl. Tabelle 2). Im Vergleich zu Japan, Großbritannien und Frankreich kann Deutschland sich jedoch behaupten. Erstaunlich ist auch die Patentleistung der Schweiz, die in Relation zur Bevölkerung und zum BIP noch weit stärker als die der USA ist. Auf die überragende Stellung der Novartis AG wurde bereits hingewiesen.

Die US-Universitäten tragen 3,8 % der gesamten Ankerpatente und 3,4 % der Elternpatente bei. Deutsche Universitäten haben keinerlei Ankerpatente hervorgebracht und nur 0,07 % der gesamten Elternpatente. Japanische Universitäten haben 0,02 % der Elternpatente beigesteuert, aber ebenfalls keine Ankerpatente. Aus den Schweizer Universitäten kommen weder Anker- noch Elternpatente.

Die Patentschwäche des deutschen Wissenschaftssystems an einer Stelle, an der Patente von herausragender Bedeutung sind, nämlich bei der Zulassung von Medika-

1 Jedes Ankerpatent (und im Folgenden auch Elternpatent) wurde jeweils nur einmal einer Nation zugeordnet. Eine Anmeldung einer US-Universität gemeinsam mit einem US-Unternehmen geht mit nur einer Patentanmeldung der USA einher.

2 Das National Institute of Health der USA hat keine Patente angemeldet.

Abbildung 3
**Zahl der Ankerpatentanmeldungen**

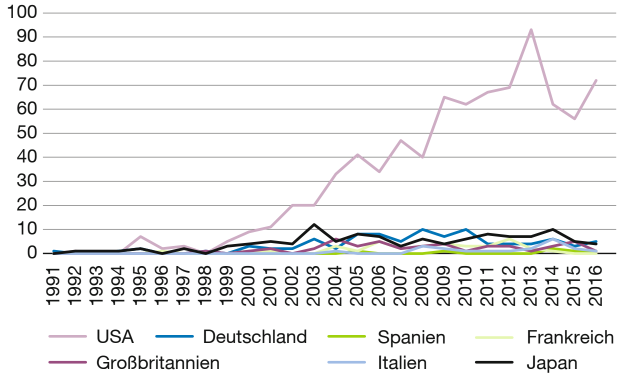

Quelle: eigene Darstellung auf Basis von FDA.

menten, mag der Tatsache geschuldet sein, dass deutsche Universitäten ihre Schwerpunkte eher in der Lehre und der Grundlagenforschung sehen. An den Unikliniken und den lebenswissenschaftlichen Instituten wird die Translation zwar nicht behindert, aber auch nicht gefördert, indem etwa Leistungen auf diesem Gebiet bei Berufungen oder Gehaltsfestsetzungen eine besondere Rolle spielen. Die zahlreichen Empfehlungen, unter anderem der Deutschen Forschungsgemeinschaft (DFG, 2004) zur sogenannten „leistungsorientierten Mittelvergabe“ sind an vielen Stellen nach Jahrzehnten nicht umgesetzt. In der Außendarstellung schreiben sich Universitäten zwar manchmal, wie im Fall von Biontech, Leistungen prominenter Forschender zu. Verwertungsvereinbarungen mit Pharmaunternehmen aber haben sie nur in seltenen Fällen geschlossen, obwohl ihnen nach dem Arbeitnehmererfindergesetz, sofern ihre Zurechnung zutrifft, auch die Rechte an den Forschungsergebnissen ihrer Beschäftigten zuständen.

## Zur Innovationskraft der deutschen außeruniversitären Forschungseinrichtungen

Tabelle 1 impliziert, dass die außeruniversitären Forschungsinstitutionen die Innovationslücke Deutschlands, speziell im Vergleich zu den USA nicht schließen können. Tabelle 2 detailliert den Beitrag der einschlägigen deutschen außeruniversitären Forschungsinstitutionen zu den Anker- und Elternpatenten, die den NME zugrunde liegen und vergleicht ihren Output mit den US-Universitäten. Die Max-Planck-Gesellschaft (MPG) nimmt mit 8 Ankerpatenten hinter der (überragenden) University of California und der University of Texas den dritten Platz ein. Das Deutsche Krebsforschungszentrum (DKFZ, 6 Elternpatente), in dem sich immerhin 2.800 Beschäftigte mit einem Budget von jährlich mehr als 250 Mio. Euro der translationalen Krebsforschung widmen (Puchta, 2016) und die Fraunhofer-Gesellschaft (FG, 5 Elternpatente) können kein einziges Ankerpatent vorweisen. Der FG muss man zugutehalten, dass nur fünf der 80 Institute und Einrichtungen in den hier relevanten Bereichen der Biologie und Medizin tätig sind.

Für eine Ermittlung der Pharmaforschungseffizienz der Institutionen wäre es angemessen, den Pharma-Patent-Output in Relation zu den Etats der Pharmaforschungsfachbereiche zu setzen. Unsere entsprechenden Anfragen an die deutschen außeruniversitären Forschungsinstitute blieben jedoch unbeantwortet. Die in Spalte (3) der Tabelle 2 gelisteten kaufkraftbereinigten Budgets für FuE-Ausgaben der Forschungsinstitutionen von 2002 bis 2018 dürfen nicht zu Berechnungen eines Effizienzmaßstabs genutzt werden, da die Budgets (je nach Forschungsausrichtung) in unterschiedlichem Maße auch für andere Forschungsbereiche wie z. B. die Physik verwendet wurden. Über die größten Budgets verfügen die University of California und (mit einem gewissen Abstand) die Universität Texas, die Johns Hopkins University, die FG und die MPG.

Zu unserer Analyse mag es Einwände geben: Sollten bestimmte Länder bzw. Firmensitze komparative Vorteile beim Anmelden von NME oder Anker- und Elternpatenten haben (etwa aufgrund erfahrungsbedingter, finanziell oder sonstig begründeter Vorteile), so benötigten die Daten eine Korrektur. Wir fanden hierfür jedoch wenig Anhaltspunkte. Es existieren zwar z. B. zwei NME-Anmeldungen durch Novartis Pharmaceuticals Corporation (East Hanover, USA), 14 weitere jedoch durch Novartis AG (Basel, CH), was dagegenspricht, dass der Novartis-Konzern vorzugsweise durch seine US-amerikanische Niederlassung anmelden lässt.

Der Einschätzung der NME als harter Kern des weltweiten medizinischen Fortschritts könnte entgegengehalten werden, dass dabei Fortschritte im Gesundheitswesen vernachlässigt werden (z. B. bei Diagnose-, Versorgungs-, und Präventionsmaßnahmen), die auf der Anwendung bekannter Techniken basieren oder auf Prozessinnovationen, die zu geringeren Kosten, höherer Qualität oder geringerer Durchlaufzeit führen. Auch könnte angeführt werden, dass biomedizinische Innovationen in der klinischen und in der Grundlagenforschung vernachlässigt werden. Die Position Deutschlands in der messbaren Grundlagenforschung zeigt jedoch Parallelen zu den Ergebnissen der NME-Analysen. Im Index von Nature (2021), der den Publikationsoutput der Länder in 82 naturwissenschaftlichen Fachzeitschriften misst, die von einem unabhängigen Gremium führender Forscher:innen nach Reputation ausgewählt werden, belegt Deutschland – bezogen auf alle naturwissenschaftlichen Veröffentlichungen zwischen 1. Dezember 2019 und 30. November 2020 einen vierten Platz (vgl. Tabelle 4). In den Life Sciences ist Deutschland mit einem Veröffentlichungsanteil von 10,2 % auf Platz zwei.

Tabelle 1

**NME, Anker- und Elternpatente ausgewählter Nationen**

| | NME | | | Ankerpatente | | | Elternpatente | | |
|---|---|---|---|---|---|---|---|---|---|
| | absolut | je 1 Mio. Einwohner | je 1 Mio. US-$ BIP | absolut | je 1 Mio. Einwohner | pro Mio. US-$ BIP | absolut | je 1 Mio. Einwohner | je 1 Mio. US-$ BIP |
| USA | 207 | 0,63 | 9,89 | 860 | 2,62 | 41,09 | 13.731 | 41,84 | 656,04 |
| Universitäten | – | – | – | 53 | 0,16 | 2,53 | 1.018 | 3,10 | 48,64 |
| staatlich finanzierte Forschungsinstitute | – | – | – | 20 | 0,06 | 0,96 | 28 | 0,09 | 1,34 |
| Japan | 18 | 0,14 | 3,56 | 117 | 0,93 | 21,17 | 2.845 | 22,55 | 563,37 |
| Universitäten | – | – | – | – | – | – | 7 | 0,06 | 1,39 |
| staatlich finanzierte Forschungsinstitute | – | – | – | – | – | – | – | – | – |
| Deutschland | 34 | 0,41 | 8,95 | 97 | 1,17 | 25,53 | 2.573 | 30,99 | 677,11 |
| Universitäten | – | – | – | 0 | – | – | 21 | 0,25 | 5,53 |
| staatlich finanzierte Forschungsinstitute | – | – | – | 8 | 0,10 | 2,11 | 49 | 0,59 | 12,89 |
| Großbritannien | 11 | 0,16 | 4,06 | 53 | 0,78 | 19,56 | 1.411 | 20,78 | 520,66 |
| Universitäten | – | – | – | 1 | 0,01 | 0,37 | 18 | 0,27 | 6,64 |
| staatlich finanzierte Forschungsinstitute | – | – | – | – | – | – | – | – | – |
| Frankreich | 4 | 0,06 | 1,54 | 36 | 0,56 | 13,85 | 868 | 13,40 | 333,85 |
| Universitäten | – | – | – | – | – | – | 15 | 0,23 | 5,77 |
| staatlich finanzierte Forschungsinstitute | – | – | – | – | – | – | 40 | 0,62 | 15,38 |
| Schweiz | 26 | 3,02 | 34,67 | 71 | 8,25 | 94,67 | 103 | 11,96 | 137,33 |
| Universitäten | – | – | – | – | – | – | – | – | – |
| staatlich finanzierte Forschungsinstitute | – | – | – | – | – | – | – | – | – |
| China | – | – | – | – | – | – | 181 | 0,13 | 12,30 |
| Universitäten | – | – | – | – | – | – | 13 | 0,01 | 0,88 |
| staatlich finanzierte Forschungsinstitute | – | – | – | – | – | – | – | – | – |
| Indien | 3 | 0,00 | 1,11 | – | – | – | 153 | 0,11 | 56,46 |
| Universitäten | – | – | – | – | – | – | – | – | – |
| staatlich finanzierte Forschungsinstitute | – | – | – | – | – | – | – | – | – |
| Israel | – | – | – | 3 | 0,32 | 7,50 | 98 | 10,55 | 245,00 |
| Universitäten | – | – | – | – | – | – | 18 | 1,94 | 45,00 |
| staatlich finanzierte Forschungsinstitute | – | – | – | – | – | – | – | – | – |

Quelle: FDA.

Allerdings relativiert sich diese Position, wenn die französischen und britischen Anteile von 9,3 % bzw. 9,1 % an deren deutlich kleineren Bevölkerungszahlen gespiegelt werden. Die im Nature-Ranking verringerte Dominanz der USA (bei gleichzeitig vergrößerter Bedeutung Chinas) kann als bessere Fähigkeit der USA interpretiert werden, aus Grundlagenforschung bahnbrechende Therapeutika zu entwickeln. Ansonsten sehen wir in Verweisen auf andere medizinisch-pharmazeutische Innovationen die Gefahr einer Ausflucht ins „Nicht-Messbare“, mit der man sich einer offenen Bestandsaufnahme sowie notwendigen Reformen entzieht.

## Fazit und forschungspolitische Fragen

Robuste Leistungsindikatoren sind für einen effizienten Ressourceneinsatz in der Forschungspolitik wichtig. Wir leisten einen Beitrag bei der Erstellung besserer Leistungsindikatoren für den Bereich der Lebenswissenschaften (Medizin, Biologie, Pharmazie), indem wir die regionale Herkunft der NME untersuchen. Derartige NME leisten einen zusätzlichen und nachweisbaren Beitrag zur Verringerung menschlichen Krankenleidens, weshalb sie als herausragendes Kriterium für Innovationen auf dem Gebiet der Lebenswissenschaften gelten. NME sind selten; von 2009 bis 2019 waren es nur 378 Arzneimittel, im Schnitt weniger als 40 Zulassungen pro Jahr. Da diese Medikamente meist auch bei europäischen und asiatischen Genehmigungsbehörden eingereicht wurden und andererseits sich kaum Pharmafirmen den lukrativen amerikanischen Markt entgehen lassen, repräsentieren NME die Speerspitze des globalen medizinischen Fortschritts praktisch vollständig.

Tabelle 2
**FuE-Ausgaben und Patentanmeldungen**

| Institut | Zahl NME-genutzter Anker-patente | Zahl der in NME-Anker-patenten zitierten Elternpatente | Summe FuE-Ausgaben 2002 bis 2018 (in 1.000 US-$) |
|---|---|---|---|
| University of California | 14 | 147 | 79.437.520 |
| University of Pennsylvania | 10 | 33 | 14.332.272 |
| Max-Planck-Gesellschaft | 8 | 35 | 29.902.889 |
| Massachusetts Institute of Technology | 8 | 23 | 12.517.765 |
| University of Michigan | 4 | 37 | 26.328.591 |
| University of Pittsburgh | 4 | 22 | 12.122.742 |
| Emory University | 3 | 40 | 8.238.593 |
| Tufts College | 3 | 28 | 2.618.292 |
| University of Texas | 0 | 48 | 39.706.442 |
| Johns Hopkins University | 0 | 42 | 32.431.643 |
| University of New York | 0 | 39 | 24.386.295 |
| Harvard University | 0 | 31 | 11.961.522 |
| Leland Stanford Junior University | 0 | 29 | 14.211.468 |
| Deutsches Krebs-forschungszentrum | 0 | 6 | 2.592.996 |
| Universität des Saarlandes | 0 | 4 | – |

Quelle: eigene Zusammenstellung.

Tabelle 3
**Satzungen verschiedener Institute und Universitäten**

| Institut | Träger-schaft | Grundla-genfor-schung | For-schung | Bil-dung | Innova-tion |
|---|---|---|---|---|---|
| Helmholtz-Gemeinschaft Deutscher Forschungszentren | öffentlich | x | x | x | |
| Max-Planck-Gesellschaft zur Förderung der Wissenschaften | öffentlich | x | | | |
| Fraunhofer-Gesellschaft zur Förderung der angewandten Forschung | öffentlich | | x | | |
| Johns Hopkins University | privat | | x | x | x |
| University of California | öffentlich | | x | x | x |
| Leland Stanford Junior University | privat | | x | x | |
| Massachusetts Institute of Technology | privat | | x | x | x |
| Harvard University | privat | | | x | |
| University of Pittsburgh | öffentlich | | x | x | x |
| University of Pennsylvania | privat | | x | x | x |
| Emory University | privat | | x | x | x |
| Tufts College | privat | | x | x | x |
| National Institutes of Health | öffentlich | x | x | x | |

Wir berücksichtigen ferner die zeitlich und inhaltlich vorausgehenden Anker- und Elternpatente, deren Erkenntnisse zur Entwicklung der NME beigetragen haben. Unsere Untersuchung hat gegenüber den üblichen Summierungen der angemeldeten oder auch der registrierten Patente den Vorteil, dass sie ausschließlich Patente zählt, die von den technologischen Durchbrüchen genutzt werden und die Patente einem expliziten Werthaltigkeitstest unterzieht.

Von 2009 bis 2019 zeigt die Auswertung erhebliche Produktivitätsunterschiede zwischen verschiedenen nationalen Wissenschaftssystemen. Die Pharmaunternehmen (und in geringerem Umfang die Universitäten) der USA sind die mit Abstand überragenden Innovationsträger. Rund 55 % der NME und rund 62 % der Ankerpatente werden von ihnen angemeldet. Die USA mit ihren rund 328 Mio. Einwohner:innen produzieren mehr Pharmainnovationen als der Rest der Welt mit etwa 7,77 Mrd. Menschen.

Die Ergebnisse für Deutschland sind durchwachsen. In absoluten Zahlen erreicht Deutschland bei allen Patenten nur einen Bruchteil der US-amerikanischen Werte. Am besten schneidet noch die Industrie mit immerhin 34 Zulassungen ab, was 16 % des US-amerikanischen Wertes (207) entspricht. Bei den Ankerpatenten entfallen dagegen nur 97 Schutzschriften auf Erfinder:innen mit Wohnsitz in Deutschland, was bezogen auf die Zahl der US-Patente nur einem Anteil von 11 % entspricht. Auch bereinigt um die Bevölkerungszahl bleibt der Rückstand beachtlich.

Die US-Universitäten tragen 3,8 % der gesamten Ankerpatente und 3,4 % der gesamten Elternpatente bei. Deutsche Universitäten haben keinerlei Ankerpatente und 0,07 % der Elternpatente hervorgebracht. Die außeruniversitären Forschungsinstitutionen können die Pharmapatentlücke Deutschlands im Vergleich zu den USA nicht schließen. MPG, FG und weitere steuern deutlich weniger zu pharmazeutischen Innovationen bei als die US-Universitäten.

In der forschungspolitischen Diskussion dürfte zunächst argumentiert werden, dass Pharmaunternehmen, außeruniversitäre Forschungsinstitute und Universitäten unterschiedliche Zielsetzungen verfolgen. Universitäten haben neben der Forschung Aufgaben im Bereich der Lehre, und die deutsche Wissenschaftspolitik räumt den Universitäten auch keinen wesentlichen Platz in der Spitzenforschung ein: „Eine Besonderheit Deutschlands besteht darin, dass ein großer Teil der Spitzenforschung gar nicht an Universitäten, sondern an außeruniversitären Einrichtungen stattfindet“ (Deutscher Bundestag, 2020).

Die deutschen außeruniversitären Forschungsinstitute mit öffentlicher Trägerschaft sollen Grundlagenforschung betreiben (vgl. Tabelle 3), was ebenfalls den Patentoutput verringern kann (Aghion und Jaravel, 2015). Es fällt z. B. auf, dass die MPG sich offiziell ausschließlich der Grundlagenforschung verschreibt. Dennoch führen die Aktivitäten der MPG auch zu Patenten im Pharmabereich. Eine explizite satzungsmäßige Behinderung von Patentaktivitäten liegt offensichtlich nicht vor.

Auch an mangelnden Finanzierungsvolumen liegt es kaum. Mehr als 105 Mrd. Euro wurden 2018 in Deutschland für FuE ausgegeben, darunter 18,6 Mrd. für FuE an Hochschulen (Destatis, 2020). Die Gesamtausgaben des Bundes für FuE lagen 2019 bei 19,6 Mrd. Euro. Insgesamt 2,68 Mrd. Euro entfielen auf den Bereich der Gesundheitsforschung und -wirtschaft (BMWi, 2020). Die deutschen öffentlichen FuE-Ausgaben sind seit 2009 stärker gestiegen als in den meisten vergleichbaren Staaten (Expertenkommission Forschung und Innovation, 2021).

Die deutschen außeruniversitären Forschungsinstitutionen wie MPG, Helmholtz, Leibniz und FG genießen relativ hohe, weitgehend im Wachstum verstetigte und direkte Zuwendungen aus dem Bundeshaushalt von derzeit jährlich ca. 5,8 Mrd. Euro. Die relativ geringeren DFG-Mittel (2020 ca. 1,8 Mrd. Euro, Gesetz über die Feststellung des Bundeshaushaltsplans für das Haushaltsjahr 2020) werden hingegen in einem offenen Wettbewerb zwischen Universitäten vergeben. Anders ist die Situation in den USA: Das dortige National Institutes of Health (NIH) fungiert primär als qualifizierter Geldverteiler, der Dritten jährlich rund 40 Mrd. US-$ für medizinische Forschung zur Verfügung stellt. Die Finanzmittel fließen zu mehr als 80 % an universitäre Akteure. Statt wie in Deutschland das Gros der Bundesmittel in „bundeseigene“ Institute zu leiten, fördert das NIH den Wettbewerb unter vielen Dritten. Nur 10 % der NIH-Mittel fließt in eigene Labore auf dem NIH Campus (NIH, 2014a).[3]

In Deutschland dürfte es weniger um das weitere Wachstum, sondern eher um die Effizienz der Forschungsförderung gehen. Hierbei sollte die Forschungsförderung in Deutschland in Richtung eines offenen Wettbewerbs gehen und die Arbeitsteilung zwischen den deutschen Universitäten und den außeruniversitären Forschungsinstitutionen einer Prüfung auf Aktualität unterzogen werden.[4]

Tabelle 4

**Nature-Index in naturwissenschaftlichen Fachzeitschriften, 1.12.2019 bis 30.11.2020**

| | Alle Bereiche (%) | Life Science (%) |
|---|---|---|
| USA | 29,4 | 37,3 |
| China | 16,6 | 7,9 |
| Frankreich | 10,6 | 9,3 |
| Deutschland | 8,3 | 10,2 |
| Großbritannien | 6,2 | 9,1 |
| Japan | 3,4 | 2,5 |
| Schweiz | 2,2 | 2,4 |
| Israel | 0,7 | 0,8 |
| Indien | 0,4 | 0,0 |

Quelle: Nature (2021); eigene Berechnungen.

Von zentraler Bedeutung ist eine Stärkung der Patentaktivitäten durch die Restrukturierung der Forschungsbedingungen in Deutschland, in der die Wissenschafts- und Wertekultur in Richtung einer höheren Bedeutung von Patenten beeinflusst wird. Bezüglich der außeruniversitären Forschungsinstitute sollte der Pakt für Forschung und Innovation (BMBF, 2021) für eine verbesserte Translation geschärft werden. An den deutschen Universitäten sollte die begrenzte Rolle von Patenten bei der Berufungs- und Evaluierungspolitik hinterfragt werden.

Zudem sollte darüber nachgedacht werden, „die Bereitschaft zu Gründungen und vor allem zu Ausgründungen aus dem Wissenschaftssystem heraus zu befördern“ (Wissenschaftsrat, 2021). Auch Ideen und Konzepte wie die einer European Biomedical Research and Development Agency BARDA (European Parliament, 2021), einer pharmazeutischen Innovationsagentur (BMBF, 2021) oder dem Netzwerk Universitätsmedizin (2021) können zielführend sein, solange in ihren Anreizmechanismen eine hinreichend starke Orientierung auf sinnvolle Outputvariablen gelingt. Eine Orientierung auf Inputvariablen wie Drittmittel oder auf weniger geeignete und im Übrigen hinreichend geförderte Outputvariablen wie Veröffentlichungen, Konferenzen etc. dürfte weniger zielführend sein.

3 Auch die Analyse der institutionellen, regulativen, bildungspolitischen, militärpolitischen und steuerlichen Vorteile von Pharmaforschung in den USA sollte beachtet werden. Diese fundamentalen Unterschiede dürften jedoch allenfalls langfristig beeinflussbar sein.

4 Die gewachsenen Strukturen basieren letztlich auf der Gründung der Kaiser-Wilhelm-Gesellschaft zur Förderung der Wissenschaften e. V. 1911, deren Institute 1953 in die heutige MPG übernommen wurden.

## References

[CR1] Aghion P, Jaravel X (2015). Knowledge Spillovers, Innovation and Growth. The Economic Journal.

[CR2] BMBF (Bundesministerum für Bildung und Forschung) (2021), Pakt für Forschung und Innovation, https://www.bmbf.de/de/pakt-fuer-forschung-und-innovation-546.html (2. Mai 2021)

[CR3] BMWi (2020), Interne Ausgaben für Forschung und Entwicklung: Deutschland, Jahre, Einrichtungsart, https://www.datenportal.bmbf.de/portal/de/K1.html (2. Mai 2021).

[CR4] Budish E, Roin B N, Williams H (2015). Do Firms Underinvest in Long-Term Research? Evidence from Cancer Clinical Trials. American Economic Review.

[CR5] Destatis (2020), Interne Ausgaben für Forschung und Entwicklung nach Sektoren und Berichtsjahren https://www.destatis.de/DE/Themen/Gesellschaft-Umwelt/Bildung-Forschung-Kultur/Forschung-Entwicklung/Tabellen/forschung-entwicklung-sektoren.html;jsessionid=B1AEFF432A536B8696FE859C0122D415.internet712 (20. April 2021).

[CR6] DFG (2004), Empfehlungen zu einer leistungsorientierten Mittelvergabe (LOM) an den Medizinischen Fakultäten, Stellungnahme der Senatskommission für Klinische Forschung der Deutschen Forschungsgemeinschaft, https://www.dfg.de/download/pdf/dfg_im_profil/reden_stellungnahmen/2004/stellungnahme_klinische_forschung_04.pdf (2. Mai 2021).

[CR7] Deutscher Bundestag (2020), Zu Lizenzerträgen aus Patentierungen an Hochschulen, Wissenschaftliche Dienste, https://www.bundestag.de/resource/blob/691798/fb1202fa2a5e1937eb403b8b0ebae3f9/WD-8-016-20-pdf-data.pdf (5. Mai 2021).

[CR8] Emory College of Arts and Sciences (2020), Mission Statements, http://catalog.college.emory.edu/about/mission.html (5. Mai 2021).

[CR9] European Parliament (2021), Legislative Proposal to Establish a European Biomedical Research and Development Agency (BARDA), https://www.europarl.europa.eu/legislative-train/theme-promoting-our-european-way-of-life/file-european-biomedical-research-and-development-agency (4. Juli 2021).

[CR10] Expertenkommission Forschung und Innovation (2021), Gutachten zu Forschung, Innovation und technologische Leistungsfähigkeit Deutschlands, https://www.e-fi.de/fileadmin/Assets/Gutachten/2021/EFI_Gutachten_2021.pdf (20. April 2021).

[CR11] FDA (2020), New Drugs at FDA: CDER’s New Molecular Entities and New Therapeutic Biological Products, https://www.fda.gov/drugs/development-approval-process-drugs/new-drugs-fda-cders-newmolecular-entities-and-new-therapeutic-biological-products (18. April 2021).

[CR12] Fraunhofer-Gesellschaft (2015), Satzung, https://www.fraunhofer.de/content/dam/zv/de/ueber-fraunhofer/Satzung-Fraunhofer-Gesellschaft.pdf (18. April 2021).

[CR13] Grabowski H (2002). Patents, innovation and access to new pharmaceuticals. Journal of International Economic Law.

[CR14] Harvard College (2020), Mission, Vision & History, https://college.harvard.edu/about/mission-vision-history (4. Mai 2021).

[CR15] Helmholtz-Gemeinschaft Deutscher Forschungszentren (2018), Satzung, https://www.helmholtz.de/fileadmin/user_upload/03_ueber_uns/organisation/satzung/Satzung_Helmholtz-Gemeinschfat-e.V.pdf (5. Mai 2021).

[CR16] Johns Hopkins University (2020), History & Mission, https://www.jhu.edu/about/history/ (26. Mai 2021).

[CR17] Massachusetts Institute of Technology (2020), What’s MIT’s mission statement?, https://mitadmissions.org/help/faq/mit-mission-statement/ (4. Mai 2021).

[CR18] Max-Planck-Gesellschaft zur Förderung der Wissenschaften (2012), Satzung der Max-Planck-Gesellschaft zur Förderung der Wissenschaften.

[CR19] Nagaoka S, Motohashi K, Goto A (2010). Patent Statistics as an Innovation. Handbook of the Economics of Innovation.

[CR20] Nature (2021), nature index, Institutions Output, https://www.natureindex.com/institution-outputs/generate/All/global/All/n_article (2. Mai 2021).

[CR21] Netzwerk Universitätsmedizin (2021), Uniklinika und Politik im Verbund, https://www.netzwerk-universitaetsmedizin.de/organisation (6.Juli 2021).

[CR22] NIH (National Institutes of Health) (2014a), Budget, https://www.nih.gov/about-nih/what-we-do/budget (18. April 2021).

[CR23] NIH (National Institutes of Health) (2014b), Mission and Goals, https://www.nih.gov/about-nih/what-we-do/mission-goals (17. April 2021).

[CR24] OECD (2008), OECD Patent Statistics Manual, OECD Publishing.

[CR25] Sampat B N, Lichtenberg F R (2011). What are the respective roles of the public and private sectors in pharmaceutical innovation?. Health affairs.

[CR26] Scherer F M, Harhoff D (2000). Technology policy for a world of skew-distributed outcomes. Research Policy.

[CR27] Stanford University (2020), Stanford’s Mission, https://web.stanford.edu/dept/registrar/bulletin1112/4792.htm (16. April 2021).

[CR28] Trajtenberg M, Henderson R, Jaffe A (1997). University versus corporate patents: A window on the basicness of invention. Economics of Innovation and new technology.

[CR29] Tufts University (2020), Mission, Vision & Themes, https://www.tufts.edu/about/mission-vision (4. Mai 2021).

[CR30] University of California (2020), UC’s Mission, https://www.ucop.edu/ucmission/ (16. April 2021).

[CR31] University of Pennsylvania (2020), A Tradition of Excellence, https://www.upenn.edu/about (26. Mai 2021).

[CR32] University of Pittsburgh (2020), Mission and Vision Statements, https://www.comm.pitt.edu/about/mission-and-vision-statements (16. April 2021).

